# Comparing emotion regulation, perceived discrimination, perceived social support and anxiety in North African immigrant and native French populations

**DOI:** 10.1038/s41598-026-58155-0

**Published:** 2026-07-03

**Authors:** Rania Driouach, Constantina Badea, Sylvia Martin

**Affiliations:** 1 Université Paris Nanterre, Laboratoire Parisien de Psychologie Sociale, Paris, France; 2 Faculty of Medicine and Pharmacy, Center for Research and Bioethics, Uppsala University, Uppsala, Sweden

**Keywords:** Perceived discrimination, Anxiety, Emotion regulation, Social support, Minority stress, North African immigrants, Cultural psychology, Mental health disparities, Health care, Psychology, Psychology

## Abstract

Discrimination is a major psychosocial stressor associated with adverse mental health outcomes among ethnic minorities. However, the mechanisms linking discrimination to anxiety remain poorly understood, particularly among North African populations in France. Drawing on Hatzenbuehler’s minority stress mediation framework, this study examined whether emotion regulation and perceived social support explain the relationship between perceived discrimination and anxiety. A total of 370 adults living in France (198 North African immigrants and 172 native French participants) completed measures of anxiety (GAD-7), perceived discrimination (InDI-M), emotion regulation (CERQ-short), and perceived social support (MSPSS). Group comparisons and mediation analyses were conducted using non-parametric tests and bootstrapping. North African participants reported higher levels of perceived discrimination than native French participants, but differences were found for anxiety or maladaptive emotion regulation. Mediation analyses showed that only among North African participants, perceived discrimination was associated with higher anxiety, partially through maladaptive emotion regulation. These findings suggest that discrimination may be associated with anxiety through emotion regulation processes rather than through differences in overall anxiety levels between groups, highlighting the importance of examining culturally and socially embedded psychological mechanisms linking discrimination to emotional well-being.

**Supplementary Information:**

The online version contains supplementary material available at 10.1038/s41598-026-58155-0.

## Introduction

In increasingly multicultural European societies, understanding how structurally embedded discrimination shapes psychological functioning among minority populations has become both a scientific and societal priority. In France, individuals of North African or Maghrebi origin (Algerian, Moroccan, Tunisian) constitute the country’s largest postcolonial minority group, numbering over four million people^1^. Their minority status is embedded within a distinct sociohistorical context shaped by colonial legacies, processes of racialization, and ongoing public debates concerning immigration, secularism, and national identity^2,3^.

Beyond their demographic significance, North African populations in France are disproportionately exposed to structural and interpersonal discrimination across multiple life domains, including employment, housing, education, and policing^4–6^. They are also overrepresented in socioeconomically disadvantaged urban neighborhoods, where structural inequalities and territorial stigmatization may compound experiences of marginalization^3^. Such contextual factors do not merely increase exposure to stressors; they may also shape how emotions are regulated and how social resources are mobilized in response to discrimination^7,8^.

Although epidemiological research indicates a relatively high prevalence of anxiety symptoms among individuals of North African origin in France^9^, the psychological processes linking discrimination exposure to anxiety remain insufficiently understood. Existing studies primarily document disparities in prevalence rates, yet fewer examine the mechanisms and dynamcocs of minority’s psychological distress. The present study addresses this gap by examining whether emotion regulation (ER) and perceived social support (PSS) help explain associations between perceived discrimination (PD) and anxiety among North African immigrants compared with native French participants.

Rather than merely documenting group differences, this study seeks to clarify how minority stress processes operate within a historically marginalized population embedded in a specific cultural and structural context.

### Anxiety, perceived discrimination and minority status in the French context

A substantial body of international research shows that members of stigmatized and minority groups are at elevated risk for anxiety and psychological^10–12^. Immigrants and their descendants frequently encounter prejudice, xenophobia, and social exclusion across the life course, and sometimes across generations^8,13^. Chronic exposure to identity-based stressors has been associated with increased emotional burden, physiological stress responses, and heightened vulnerability to mental health difficulties^14,15^.

In France, anxiety levels have increased in the general population in recent years^16–18^. However, disparities appear particularly pronounced among ethnic minorities. Individuals of North African origin report higher rates of anxiety disorders than native-born individuals (25.3% vs. 20.7%^9^; a pattern also observed among migrant populations across Europe and North America^19–22^.

North African minorities in France occupy a distinct sociocultural position shaped by racialization and, in many cases, religious stigmatization. Individuals of Maghrebi descent are often perceived simultaneously through ethnic and Muslim identity markers, potentially increasing exposure to both ethnic discrimination and Islamophobia^2,23^. Younger generations born in France may therefore experience tensions between civic belonging and perceived exclusion, which can heighten sensitivity to discrimination-related stressors^3^.

PD defined as the subjective experience of unfair treatment based on group membership (Reference^24,25^ has consistently been associated with anxiety across minority populations^14,15^. However, empirical evidence in France remains scarce. To our knowledge, only one study has documented a positive association between PD and anxiety among individuals of North African origin^26^, highlighting the need for theoretically grounded research examining the mechanisms linking discrimination to anxiety in this context.

### Integrating Hatzenbuehler’s minority stress framework

To address this gap, the present study draws on Hatzenbuehler’s (2009)^27^ psychological mediation framework, an extension of minority stress theory. Rather than conceptualizing minority status as directly causing psychological distress, this framework proposes that socially structured stigma generates identity-related stressors (e.g., prejudice events, anticipated rejection), which in turn affect mental health through disruptions in general psychological processes.

A central contribution of this model lies in its identification of mediating mechanisms, particularly ER and interpersonal functioning. Chronic exposure to discrimination may heighten emotional reactivity, increase reliance on certain regulatory strategies, and weaken perceptions of social connectedness. Over time, these processes may increase vulnerability to anxiety and depressive symptoms.

Although originally formulated in the context of sexual minority stress, the psychological mediation framework is not population-specific^27,28^. Its emphasis on universal psychological processes makes it theoretically applicable to ethnocultural minorities exposed to systemic discrimination. Applying this framework to North African populations in France allows us to examine whether similar mediational pathways operate within a postcolonial European context characterized by racialization and secular-national identity tensions.

### Emotion regulation in cultural and discriminatory contexts

ER refers to the processes through which individuals influence the experience, intensity, and expression of their emotions^29^. Strategies such as cognitive reappraisal and problem-solving are generally associated with psychological adjustment, whereas rumination, experiential avoidance, and persistent expressive suppression are frequently linked to elevated anxiety^30–32^.

However, the adaptiveness of regulatory strategies is context-dependent. In environments characterized by social threat or discrimination, strategies such as emotional restraint or suppression may serve short-term protective social functions by minimizing interpersonal conflict or reducing exposure to further stigmatization. Within collectivist cultures such as North African families restraint may be culturally valued as a means of preserving family cohesion and social harmony^33,34^. Consequently, classifying suppression as uniformly “maladaptive” risks overlooking its sociocultural meaning^35,36^.

Nevertheless, repeated reliance on high-effort regulation in response to chronic discrimination may entail cumulative psychological costs. International research suggests that minority individuals exposed to discrimination report greater ER difficulties and higher use of rumination and suppression^27,35,37–40^. These patterns are best understood not as inherent deficits but as contextually shaped responses to repeated identity-based stressors.

### Perceived social support as an interpersonal mediator

Beyond intrapersonal processes, Hatzenbuehler’s framework also highlights the importance of interpersonal mechanisms, particularly perceived social support. PSS refers to an individual’s perception of the availability and quality of support provided by their social network^41^.

Extensive research shows that PSS can both directly protect against psychological distress and buffer the impact of stressful experiences^42,43^. Lower levels of perceived support have been consistently associated with higher anxiety, whereas stronger support predicts better emotional adjustment^7^.

Among minority populations, discrimination may undermine PSS through several pathways. Experiences of rejection and stigmatization can erode trust in majority-group members and institutions, increase social withdrawal, and heighten concerns about negative evaluation^44–46^. Even when objective support is available, repeated discrimination may weaken individuals’ subjective sense of belonging and security^8^.

At the same time, family cohesion and collective identity often represent important sources of resilience in minority communities. Discrimination occurring in broader societal contexts, however, may create tensions between strong intra-community support and experiences of external exclusion^47–50^.

To date, study conducted in France has simultaneously examined ER and PSS as potential mechanisms linking PD to anxiety among adults of North African origin. Investigating these pathways may help clarify whether anxiety disparities reflect differential exposure to discrimination, regulatory processes, erosion of social resources, or a combination of these mechanisms.

### Aims and hypotheses

The first objective is to examine whether North African immigrants and native French participants differ in their levels of perceived discrimination, ER strategies, perceived social support, and anxiety symptoms.

The second objective is to investigate the pattern of associations among perceived discrimination, emotion regulation, PSS, and anxiety symptoms, with particular attention to whether psychological and interpersonal processes statistically account for the link between discrimination and anxiety. Consistent with the psychological mediation framework, the study examines whether ER strategies and PSS function as intermediary mechanisms through which PD is associated with anxiety symptoms.

#### Hypotheses

Participants of North African origin were expected to report higher levels of perceived discrimination, maladaptive ER strategies, and anxiety compared with native French participants. Across participants, higher levels of PD were expected to be associated with higher levels of anxiety and greater reliance on maladaptive ER strategies, as well as with lower levels of perceived social support. In turn, maladaptive ER strategies were expected to be positively associated with anxiety, whereas PSS was expected to be negatively associated with anxiety. Finally, consistent with the psychological mediation framework of minority stress, ER strategies and PSS were expected to statistically mediate the association between PD and anxiety.

## Method

### Participants

Participants were recruited in 2023 from January to May. Recruitment was conducted online using a link to a questionnaire hosted on the Qualtrics platform. The link was distributed via social media and shared with associations related to the target population to maximize outreach and participant diversity. The protocol adhered to Declaration of Helsinki ethics recommendations regarding patient information and collecting informed consent, and to General Data Protection Regulation (GDPR) rules regarding the collection of anonymous data. Participants provided online informed consent for both participation and the publication of results.The survey, which included standardized psychological scales and demographic questions, took approximately 15 to 30 min to complete.

Participants were eligible for inclusion if they were at least 18 years old, held French nationality or were of North African origin, resided in France, and possessed sufficient proficiency in the French language to complete the questionnaire. All participants provided informed consent prior to participation. Individuals who had never lived in France were excluded from the study. Participants were naïve as to the purpose of the study.

The total sample comprised 370 participants, including 198 individuals of North African immigrant origin and 172 native French participants. The mean age of the North African group was 29.9 years (*SD* = 10.1), significantly higher than that of the native French group (*M* = 25.5, *SD* = 10.3), *t*(368) = 4.40, *p* < 0.001. Regarding gender, women constituted the majority in both groups (73.7% among North African participants and 84.3% among native French), while men represented 25.7% and 13.9%, respectively. This difference was statistically significant, *χ*^*2*^(1, *N* = 370) = 9.70, *p* = 0.002. A small proportion of participants identified as non-binary (0.5% in the North African group and 1.7% among natives), with significant difference between groups, *χ*^*2*^(1, *N* = 370) = 1.00, *p* = 0.317. Concerning sociocultural status, 39.3% of North African participants and 61.6% of native French participants were students, *χ*^*2*^(1, *N* = 370) = 4.20, *p* = 0.039. Conversely, professional integration was more frequent among North African participants (55.5%) than among native French (31.9%), *χ*^*2*^(1, *N* = 370) = 18.30, *p* < 0.001. A small proportion of participants reported other statuses (5.0% and 6.3%, respectively), with significant difference between groups, *χ*^*2*^(1, *N* = 370) = 0.83, *p* = 0.39. However, in this research we did not examine the impact of age or gender differences inside each group. We focused our analysis on the comparison between North African immigrants and French people.

### Study procedure

The questionnaire was created on Qualtrics and distributed online through various social media platforms, including student, community, and professional groups, to obtain a diverse range of sociodemographic profiles among participants (see Supplementary file 1). The beginning of the questionnaire explained the purpose of the study, and participants were then invited to provide their informed consent to participate. Sociodemographic questions, as well as the identification of participants’ nationality, were asked at the start of the questionnaire. Afterward, participants could respond to items related to the different scales used to measure our variables of interest.

Using participants’ responses regarding their nationality and that of their ancestors, we grouped them according to nationality. The grouping criteria were inspired by the study of Pignon et al. (2018)^9^. *Participants of North African immigrant background* were defined as individuals (a) born in the Maghreb and currently residing in France (first generation), (b) born in France with at least one parent originating from the Maghreb (second generation, descendants of immigrants), or (c) born in France with at least one grandparent originating from the Maghreb (third generation, descendants of immigrants). *Native French participants* were defined as individuals born in France whose parents and grandparents were also born in France, and who therefore did not have an immigrant background.

### Materials

*Generalized Anxiety Disorder 7-item scale (GAD-7)* was used to assess anxiety symptoms. The French version of the GAD-7 is a valid and reliable instrument for general, clinical, and minority populations^51^. It includes seven items evaluating the frequency of anxiety symptoms over the past two weeks, rated on a 4-point Likert scale from 0 (“Not at all”) to 3 (“Nearly every day”). Total scores range from 0 to 21, with higher scores indicating greater anxiety severity; scores above 7 suggest possible generalized anxiety disorder. In this study, the scale showed good internal consistency (Cronbach’s α = 0.89).

*Intersectional Major Discrimination Index (InDI-M)* (InDI-M^52^, available in French translation, was used to assess experiences of major discrimination. The English version has demonstrated good validity and reliability across multiple minority populations, although psychometric validation in French has not yet been established. The scale includes 13 items (e.g., “In the past 12 months, has a healthcare provider ever refused to treat you because of who you are?”) rated on a 3-point Likert scale from 0 (“Never”) to 2 (“More than once”). Total scores range from 0 to 13, with higher scores indicating greater perceived discrimination. In this study, the scale showed acceptable internal consistency (Cronbach’s α = 0.73).

Because this measure was not validated in the French context, a confirmatory factor analysis (CFA) was conducted to examine the factorial structure of the InDI-M items in the present sample. The chi-square test was statistically significant, χ^2^(65) = 173, p < 0.001, indicating some discrepancy between the model and the observed covariance matrix. Given the sensitivity of the chi-square statistic to sample size, additional fit indices were examined.

Overall, the CFA results suggested suboptimal model fit. The comparative fit index (CFI) was 0.697, which falls below conventional thresholds for acceptable fit. The standardized root mean square residual (SRMR) was 0.072, which is below the 0.08 threshold and indicates acceptable residual fit. The root mean square error of approximation (RMSEA) was 0.088 (90% CI [0.072, 0.104]), suggesting moderate to poor model fit. Taken together, these indices indicate that the proposed factor structure was only partially supported in this sample.

To provide additional evidence relevant to construct validity, we examined the association between PD and anxiety. Consistent with theoretical expectations and previous research on minority stress, PD was positively associated with anxiety in our sample, r(212) = 0.28, p < 0.001. This relationship provides preliminary evidence of convergent validity, as experiences of discrimination are known to be associated with adverse mental health outcomes.

*Cognitive Emotion Regulation Questionnaire (CERQ)* (see French validation^53^ in its short 18-item version, was used to assess strategies such as self-blame, other-blame, catastrophizing, suppression and rumination (grouped as maladaptive ER strategies) and acceptance, positive refocusing, positive reappraisal, planning, and putting into perspective (grouped as adaptive ER strategies). In our study, Cronbach’s alpha values range from 0.68 to 0.83, with an average of 0.77, indicating an overall acceptable to good reliability. The positive reappraisal and perspective-taking subscales obtained Cronbach’s alpha coefficients of 0.68 and 0.69, respectively. A mean score of in-adaptive emotional regulation was calculated with higher score indicating higher difficulties to ER.

*Multidimensional Scale of Perceived Social Support (MSPSS)* The scale originally includes three sources of support—family, friends, and significant others each measured with four items. The French version has demonstrated good internal reliability and has previously been used with minority populations^54^. In the present study, only the family subscale was used due to the particular importance of family support in minority populations. Participants responded to each item on a 7-point Likert scale ranging from 1 (very strongly disagree) to 7 (very strongly agree). Example items include “My family really tries to help me” and “My family is willing to help me make decisions.” A mean score was computed by averaging the four items, with higher scores indicating greater perceived family support. The modified scale showed excellent internal consistency in the present sample (Cronbach’s α = 0.91).

## Results

### Statistical analysis

We used Jamovi open-source statistical analysis software (www.jamovi.org) to run our non-parametric tests. The level of significance was set to *p* < 0.05. Normality was assessed using the Shapiro–Wilk test. Most variables, including anxiety, PD, maladaptive ER strategies, and perceived social support, significantly deviated from normality (p < 0.01). These results indicate that most psychological measures in the sample were not normally distributed.

### Descriptive analysis

Detailed descriptive statistics concerning the subcomponents of maladaptive emotional regulation are presented in the Table 1.

**Table 1:** Spearsman’s correlation matrix.

**Variable**		**Anxiety**	**PD**	**A ER**	**PR**	**PIP**	**MER**	**RUM**	**DRAM**	**OB**
1. Anxiety	Spearman’s rho	—								
	p-value									
2. PD	Spearman’s rho	.275***	—							
	p-value	<.001	—							
3. Adaptive ER	Spearman’s rho	-.226**	-.072	—						
	p-value	.001	.311	—						
4. PR	Spearman’s rho	-.208**	.027	.604***	—					
	p-value	.003	.701	<.001	—					
5. PIP	Spearman’s rho	-.174*	-.056	.756***	.389***	—				
	p-value	.014	.436	<.001	<.001	—				
6. Maladaptive ER	Spearman’s rho	.391***	.223**	.034	-.222**	-.005	—			
	p-value	<.001	.002	.636	.002	.946	—			
7. Rumination	Spearman’s rho	.299***	.248***	.031	-.234***	.008	.627***	—		
	p-value	<.001	<.001	.668	<.001	.911	<.001	—		
8. Dramatization	Spearman’s rho	.423***	.211**	-.150*	-.232***	-.169*	.699***	.472***	—	
	p-value	<.001	.003	.035	<.001	.018	<.001	<.001	—	
9. Other-Blame	Spearman’s rho	.232**	.315***	-.192**	-.143*	-.110	.502***	.211**	.454***	—
	p-value	.001	<.001	.007	.044	.123	<.001	.003	<.001	—
10. PSS	Spearman’s rho	-.220**	-.173*	.141*	.157*	.129	-.286***	-.203**	-.180*	-.131
	p-value	.002	.015	.048	.027	.070	<.001	.004	.011	.066

PD = Perceived Discrimination; ER = Emotion Regulation; Adaptive ER = Adaptive Emotion Regulation; PR = Positive Reappraisal; PIP = Putting into Perspective; MER = Maladaptive Emotion Regulation; RUM = Rumination; DRAM = Dramatization; OB = Other-Blame; PSS = Perceived Social Support. * : p < 0.05—** : p < 0.01—***: p < 0.001.

In order to examine the differences between French people and North African immigrants, we conducted Mann–Whitney U tests due to non-normal distributions. Results showed that North African immigrants perceived higher level of discrimination compared to French people (*M* = 4.37, *SD* = 4.09 *vs M* = 3.42, *SD* = 3.32), *t* (368) = 2.42, *p* = 0.016, *d*_Cohen_ = 0.25. The difference between groups was not significant for anxiety, (*M* = 8.03, *SD* = 5.57 *vs M* = 8.05, *SD* = 5.51), *t* (368) = 0.03, *p* = 0.971, *d*_Cohen_ = 0.00, or maladaptive emotional regulation, (*M* = 2.39, *SD* = 0.75 *vs M* = 2.50, *SD* = 0.75), *t* (368) = 1.37, *p* = 0.171, *d*_Cohen_ = 0.14. Other detailed comparisons between groups are presented in the Table 2.

**Table 2:** Comparison of variables between groups.

Variables	Participants (N = 370)		North African immigrant (n = 198)		Native French (n = 172)		Mann Whitney’s U	Significance	Rank biserial correlation
	Mean	Standard error	Mean	Standard error	Mean	Standard error			
Age	27.9	9.7	29.9	10.18	25.53	10.34	23,198	<.001***	0.36
Gender: Female	78.16	–	73.73	–	84.31	–	*–*	*–*	*–*
Gender: Male	20.27	–	25.75	–	13.95	–	*–*	*–*	*–*
Gender: Non-binary	1.08	–	0.50	–	1.74	–	*–*	*–*	*–*
Sociocultural level (%) Student	49.79	–	39.38	–	61.72	–	–	–	*–*
Sociocultural level (%) Employed	44.59	–	55.61		31.96	–	–	–	*–*
Sociocultural level (%) Other	5.61	–	5.01	–	6.32	–	–	–	*–*
Anxiety	8.03	5.53	8.02	5.57	8.04	5.51	16,903	.903	−0.01
Perceved discrimination	3.92	3.78	4.36	4.09	3.41	3.32	19,307	.025*	0.13
Adaptive emotion regulation	2.97	0.76	2.99	0.79	2.96	0.72	17,333.5	.766	0.01
Positive refocusing	2.46	1.14	2.49	1.09	2.43	1.21	17,815.5	.43	0.04
Refocusion on planning	3.12	1.13	3.25	1.09	2.96	1.16	19,551	0.01**	0.14
Acceptance	2.96	1.21	2.86	1.23	3.07	1.16	15,351	0.10	−0.09
Positive reapraisal	3.43	1.09	3.43	1.09	3.42	1.11	17,195	0.87	0.01
Putting into perspective	2.92	1.05	2.92	1.02	2.91	1.09	17,403	.712	0.02
Maladaptive emotion regulation	2.44	0.75	2.16	0.51	2.22	0.54	1548	.293	−0.08
Self-Blame	2.31	1.18	2.09	1.13	2.56	1.19	12,831	<.001***	−0.24
Other-Blame	2.12	1.03	2.15	1.04	2.09	1.01	17,481	.651	0.02
Rumination	3.09	1.05	3.02	1.03	3.17	1.08	15,667	.181	−0.08
Expressive suppression	4.05	1.47	4.25	1.44	3.81	1.47	19,864	.006**	0.16
Substance use	1.48	0.88	1.29	1.69	1.69	1.01	12,850	<.001***	−0.24
Perceived social support	4.71	1.51	4.66	1.46	4.74	1.56	16,316.5	.488	−0.04

* : p < 0.05— ** : p < 0.01— ***: p < 0.001.

### Mediation test

In this short note we examine mainly the perceived discrimination, the level of anxiety and of maladaptive emotional regulation, and their links among North African immigrants.

To test whether the relationship between PD and anxiety among North African immigrants is mediated by maladaptive emotion regulation, we conducted a mediation analysis using the bootstrapping procedure recommended by Hayes and Preacher (2014). Age, gender, and student status were included as covariates in all models. The analyses were conducted using the MedMod module in Jamovi with 1,000 bootstrap samples. The indirect effect was considered significant when the 95% bootstrap confidence interval did not include zero.

For transparency, we first report the regression paths corresponding to the mediation model (Baron & Kenny, 1986). First, we tested whether PD significantly predicted anxiety. This regression analysis showed a significant association between PD and anxiety, b = 0.42, SE = 0.09, 95% CI [0.23, 0.61], t(200) = 4.39, p < 0.001, ηp2 = 0.088. Second, we tested whether PD predicted maladaptive emotion regulation. This analysis also revealed a significant relationship, b = 0.08, SE = 0.02, 95% CI [0.04, 0.13], t(200) = 3.92, p < 0.001, ηp2 = 0.071. Finally, we tested whether the effect of PD on anxiety decreased when controlling for maladaptive emotion regulation. The effect of PD on anxiety was reduced but remained significant, b = 0.29, SE = 0.09, 95% CI [0.10, 0.47], t(199) = 3.09, p = 0.002, ηp2 = 0.046, while maladaptive ER significantly predicted anxiety, b = 1.49, SE = 0.28, 95% CI [0.93, 2.05], t(195) = 5.28, p < 0.001, ηp2 = 0.12.

We then examined the indirect effect using the bootstrapping procedure. Results indicated a significant indirect effect of PD on anxiety through maladaptive emotion regulation, b = 0.12, SE = 0.03, 95% CI [0.06, 0.20], z = 3.47, p = 0.001. This indirect pathway accounted for 32.5% of the total effect, indicating that part of the association between PD and anxiety operates through maladaptive emotion regulation. The direct effect of PD on anxiety remained significant after controlling for the mediator, b = 0.26, SE = 0.09, 95% CI [0.08, 0.44], z = 2.92, p = 0.003, accounting for 67.5% of the total effect. The total effect of PD on anxiety was also significant, b = 0.39, SE = 0.09, 95% CI [0.20, 0.57], z = 4.21, p < 0.001.

Together, these results indicate a partial mediation, whereby maladaptive ER explains a significant but limited portion of the relationship PD and anxiety among North African immigrants.

**Fig. 1:**
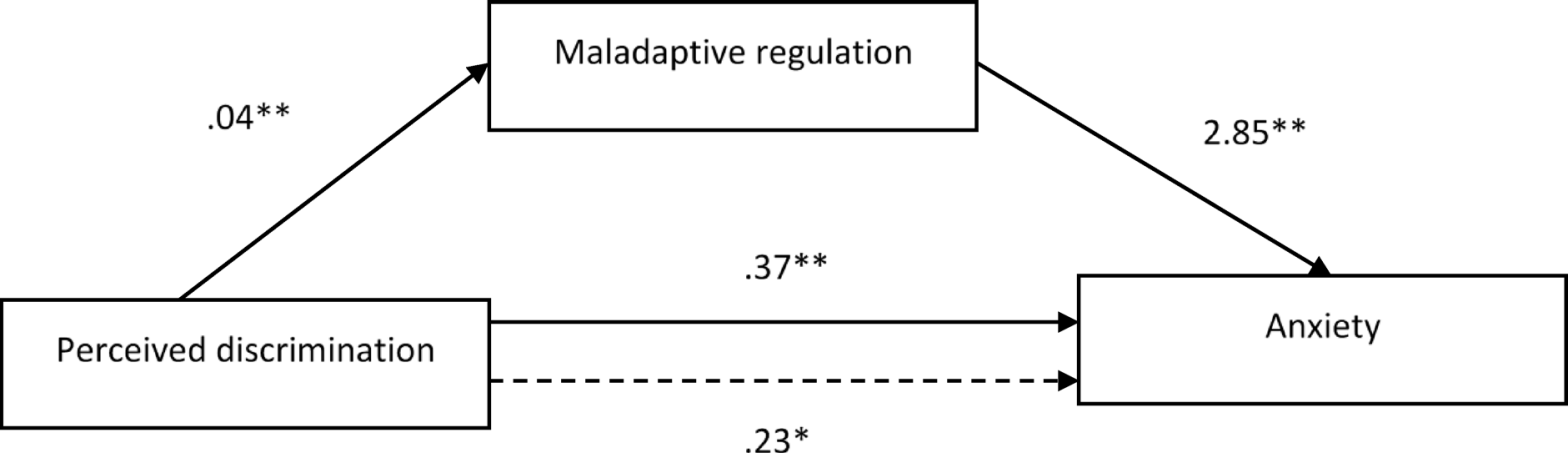
Mediation of the link between perceived discrimination and anxiety by maladaptive emotion regulation. *p* < 0.05—** : *p* < 0.01—***: *p* < 0.001.

An alternative mediation model was also tested in which the effect of PD on maladaptive ER was mediated by anxiety, while controlling for age, gender, and student status. Indeed, it is possible that PD impairs ER capacities, which in turn may increase anxiety. In this alternative model, the effect of PD on maladaptive ER decreased when anxiety was included in the model, b = 0.05, SE = 0.02, 95% CI [0.01, 0.09], t(195) = 2.42, p = 0.016, ηp2 = 0.028. Anxiety was significantly associated with maladaptive ER, b = 0.08, SE = 0.01, 95% CI [0.05, 0.13], t(195) = 5.28, p < 0.001, ηp2 = 0.15. The indirect effect in this alternative model was also significant, b = 0.03, SE = 0.009, 95% CI [0.01, 0.04], z = 3.31, p < 0.001. These findings suggest that experimental or longitudinal data would be necessary to clearly disentangle the directional relationships between PD, anxiety, and maladaptive ER (Fig. 1).

## Discussion

### Overview of the findings

The present study examined the relationships between perceived discrimination, maladaptive ER strategies, perceived social support, and anxiety among individuals of North African origin and native French participants. Drawing on Hatzenbuehler’s psychological mediation framework of minority stress^27^, the study aimed to clarify whether intrapersonal and interpersonal mechanisms may contribute to understanding how discrimination relates to psychological distress within this population.

Several findings emerge from the analyses. First, participants of North African origin reported significantly higher levels of PD compared with native French participants. However, contrary to expectations, levels of anxiety did not significantly differ between the two groups. Second, PD was positively associated with maladaptive ER strategies and anxiety, while being negatively associated with perceived social support. Third, maladaptive ER strategies were statistically associated with the relationship between discrimination and anxiety, whereas PSS did not appear as a significant mediator when ER was included in the model.

Taken together, these findings suggest that discrimination may be linked to anxiety primarily through psychological regulatory processes rather than through differences in overall anxiety prevalence between groups.

### Minority stress without mean-level anxiety differences

One notable finding of the present study is that, although North African participants reported higher levels of perceived discrimination, their levels of anxiety did not significantly differ from those of native French participants. This pattern highlights an important point emphasized in recent minority stress research: disparities in exposure to stressors do not necessarily translate into systematic differences in mean levels of mental health outcomes^15,55^.

Several explanations may account for this pattern. First, minority populations may develop coping strategies that mitigate the psychological impact of discrimination, including strong family networks or community-based support systems^56^. Second, minority stress research increasingly emphasizes heterogeneity in psychological responses to discrimination, with some individuals showing resilience despite repeated exposure to identity-based stressors^57^.

From this perspective, the absence of group differences in anxiety does not contradict minority stress theory. Rather, it suggests that the psychological consequences of discrimination may be better understood through within-person processes, such as emotion regulation, rather than solely through comparisons of mean symptom levels across groups.

### Emotion regulation as a key psychological process

Consistent with the psychological mediation framework^27^, PD was associated with greater reliance on maladaptive ER strategies, which in turn were associated with higher levels of anxiety. These findings align with a growing body of research indicating that discrimination-related stress can influence how individuals regulate their emotional experiences^30,37,38,58^.

Repeated exposure to discrimination may increase emotional vigilance and heighten negative affect, requiring individuals to mobilize regulatory strategies more frequently in everyday interactions. Over time, strategies such as rumination or persistent suppression may become habitual responses to situations perceived as socially threatening^27,37^. These regulatory patterns may contribute to sustained emotional strain and, ultimately, higher anxiety.

Importantly, these results suggest that the psychological impact of discrimination may operate less through direct effects on mental health and more through its influence on everyday ER processes.

While the present study focused primarily on maladaptive ER strategies, future research could further investigate the role of adaptive regulatory strategies in this context. In particular, it would be important to examine whether adaptive forms of ER may serve protective or buffering functions in the relationship between discrimination experiences and mental health outcomes. Such work could help clarify the conditions under which certain regulatory strategies promote resilience among individuals exposed to identity-based stressors.

### Expressive suppression, cultural norms, and racialized emotional labor

One regulatory strategy that deserves particular attention is expressive suppression. Although suppression is often conceptualized in psychological research as a maladaptive ER strategy^29,30^, its function may vary depending on cultural and social context.

Within many North African cultural contexts, emotional restraint can be socially valued as a means of preserving interpersonal harmony and maintaining respect within family and community relationships^33,34^. In such contexts, suppression may therefore serve adaptive interpersonal functions rather than reflecting emotion dysregulation.

Beyond cultural norms, expressive suppression may also be shaped by the dynamics of racialized emotional labor experienced by members of stigmatized groups. Research on racialized ER suggests that individuals belonging to minority groups often monitor and regulate their emotional expressions in interactions with majority-group members in order to avoid reinforcing negative stereotypes or escalating conflict^59–61^.

This form of emotional self-monitoring has also been described within the literature on racial battle fatigue, which highlights the cognitive and emotional effort required for individuals to navigate racially charged environments^62,63^. In such contexts, emotional restraint may represent a strategic effort to manage potentially stigmatizing social interactions. However, the sustained effort required to regulate emotional expression in these situations may become psychologically taxing over time, potentially contributing to longer-term emotional strain.

From this perspective, expressive suppression among minority individuals should not be interpreted solely as a maladaptive regulatory pattern but rather as a contextually shaped strategy embedded in both cultural norms and experiences of discrimination.

### The limited mediating role of perceived social support

Although PSS was negatively associated with anxiety, it did not appear as a significant mediator when maladaptive ER strategies were included in the model. This finding suggests that the psychological processes linking discrimination to anxiety may operate more strongly through intrapersonal regulatory mechanisms than through perceived social resources.

One possible explanation is that PSS functions primarily as a buffering factor rather than a mediating mechanism. The stress-buffering model of social support^64,65^ proposes that social relationships may mitigate the impact of stress on mental health without necessarily explaining how stress influences emotional processes.

Another possibility is that discrimination experiences occurring in broader societal contexts such as workplaces, public institutions, or encounters with authorities may not always be directly mitigated by existing support networks. In such cases, individuals may rely more heavily on internal regulatory strategies to manage the emotional consequences of these experiences.

Future research may benefit from examining more differentiated forms of social support, including family-based, peer-based, and institutional sources of support, as well as potential moderating effects of support networks on discrimination-related stress.

### Implications for minority stress research

Taken together, the present findings contribute to minority stress research in several ways. First, they extend the psychological mediation framework to the study of ethnocultural minority populations within a European context, highlighting the relevance of ER processes in understanding how discrimination relates to psychological distress.

Second, the results underscore the importance of considering the sociocultural context in which ER strategies are deployed. Strategies often labeled as maladaptive in the general population may serve adaptive social functions for individuals navigating stigmatized identities.

Finally, the findings highlight the complexity of discrimination-related stress processes. Rather than producing uniform differences in mental health outcomes between groups, discrimination may exert its influence through more subtle psychological mechanisms that shape how individuals regulate their emotional experiences in everyday social interactions.

### Limitations and future directions

Several limitations should be noted. One limitation of the present study concerns the measurement of perceived discrimination. Although we conducted a confirmatory factor analysis to examine the factorial structure of the French version of the InDI-M scale, the model showed suboptimal fit indices, suggesting that the proposed factor structure was only partially supported in this sample. While the scale demonstrated theoretically consistent associations with anxiety, providing preliminary evidence of convergent validity, the psychometric properties of the French version of the InDI-M remain insufficiently established. Consequently, caution is warranted when interpreting the magnitude of the associations observed in this study. In particular, the lack of full psychometric validation may limit the precision with which PD is captured and could affect comparisons across individuals or groups. Future research should further examine the factorial validity and reliability of the French version of the InDI-M in larger and more diverse samples to ensure the robustness of conclusions drawn from this measure.

Another limitation concerns the interpretation of the mediation analyses. Because the data are cross-sectional, the directionality of the relationships between perceived discrimination, anxiety, and maladaptive ER cannot be definitively established. Although our primary model assumed that PD contributes to maladaptive emotion regulation, which in turn increases anxiety, alternative causal pathways are also plausible. Indeed, additional analyses showed that a model in which anxiety mediates the association between PD and maladaptive ER also produced a significant indirect effect. These findings highlight that the temporal ordering of these psychological processes cannot be firmly determined using cross-sectional data. Future studies using longitudinal or experimental designs would therefore be necessary to clarify the causal relationships between perceived discrimination, ER strategies, and anxiety.

In addition, recruitment through social media may have introduced selection bias, as the French native group included more students and the North African group more employed participants. Psychiatric history, medication, and medical conditions were not controlled for, and the use of self-report measures may have introduced response biases.

### Conclusion

The present study examined the relationships between perceived discrimination, ER strategies, perceived social support, and anxiety among individuals of North African origin and native French participants. While anxiety levels did not significantly differ between groups, PD was associated with higher anxiety through ER processes.

These findings highlight the importance of examining the psychological mechanisms through which discrimination influences emotional well-being, rather than focusing solely on differences in mental health outcomes between groups. Understanding these processes may contribute to a more nuanced perspective on how minority stress operates within culturally and historically specific contexts.

## Supplementary Information

Supplementary Information.

## Data Availability

The data are available upon request to the corresponding author.
