# Supplementary material for: Comparing emotion regulation, perceived discrimination, perceived social support and anxiety in North African immigrant and native French populations
Source: Sci Rep. 2026 Jul 3;16:20549. doi: 10.1038/s41598-026-58155-0 (PMC13332036; doi:10.1038/s41598-026-58155-0)
Supplement: Supplementary file 1 — Supplementary Information. [file 41598_2026_58155_MOESM1_ESM.pdf]

Hello, I am Rania Driouach PhD student in psychology at the University of Paris Nanterre (France) and Uppsala (Sweden). I am carrying out this study resulting from my thesis under the supervision of Professor of Social Psychology Constantina Badea (University of Paris Nanterre) and Associate Professor of Medical Psychology Sylvia Martin (Uppsala University) on psychological functioning in people born in France or in other countries. If you have any questions about this research study, its procedures, or anything else you can contact me by email: 44021141@parisnanterre.fr. This questionnaire will have 6 distinct scales, its duration of administration is approximately 9 minutes. For most questions, there are no right or wrong answers, and you are free to stop whenever you want. Your responses will be anonymous and will only be used for research purposes.

Participant commitment: participation in the study consists of understanding the psychological functioning of an individual born in France and elsewhere. Participants agree to answer the investigator's questions as sincerely as possible.
Commitment of the investigator: We are committed to conducting this research in accordance with ethical and deontological provisions, to protecting the physical, psychological and social integrity of individuals throughout the research and to ensuring the confidentiality of the information collected. We are also committed to providing participants with all the support necessary to mitigate the negative effects that may arise from participating in this research.
Participant's freedom: Consent to continue research may be withdrawn at any time without giving any reason and without incurring any liability or consequences. Answers to questions are optional and failure to answer will have no consequences for the subject.
Participant information: the participant has the possibility to obtain additional information about this study from the investigator, within the limits of the constraints of the research plan.
Confidentiality of information: All information concerning participants will be kept anonymous and confidential. The computer processing is not nominative, it is therefore not included in the Data Protection Act (the right of access and rectification is not admissible). As this research is only psychological in nature, it is therefore not included in the Huriet-Sérusclat law concerning the protection of individuals in bio-medical research. The transmission of information concerning the participant for the expert opinion or for the scientific publication will also be anonymous.
Deontology and ethics: the sponsor and the investigators undertake to absolutely preserve confidentiality and professional secrecy for all information concerning the participant (Title I, articles 1, 3, 5 and 6 and title II, articles 3, 9 and 20 of the code of ethics of psychologists, France).

I agree to participate in the research
Yes
No

How old are you? (Write as a number, e.g. 23)

What is your gender?
Female

Male
Non-binary

What is your socio-professional situation?
Student/High School Student
Job seeker
Employee
Worker
Business manager
Merchant
Retired
Housewife or man
Other:

Please answer the following questions (several choices are possible)
(Indicate in which geographical area your nationality and that of your family are located)

What is your nationality ?
What is the nationality of your parents ?
What is the nationality of your grand parents ?
Answer selecting : French / Magrhebi / south saharian / North Africa (excluding maghreb) / Turkish / East Asia / Europe (excluding France) / North America / south america /other

Where do you live now? (End of block if France is selected)
Maghreb
Sub-Saharan Africa
Other:

Have you ever lived in France (End of block if not selected)
Yes
No

How long did you live in France?
Less than 1 year old
Between 1 and 2 years old
Between 2 and 3 years old
Between 3 and 5 years old
Between 5 years and older
More than 10 years

# GAD-7

| Over the <u>last 2 weeks</u>, how often have you been bothered by the following problems? *(Use "✔" to indicate your answer)* | Not at all | Several days | More than half the days | Nearly every day |
|---|---|---|---|---|
| **1.** Feeling nervous, anxious or on edge | 0 | 1 | 2 | 3 |
| **2.** Not being able to stop or control worrying | 0 | 1 | 2 | 3 |
| **3.** Worrying too much about different things | 0 | 1 | 2 | 3 |
| **4.** Trouble relaxing | 0 | 1 | 2 | 3 |
| **5.** Being so restless that it is hard to sit still | 0 | 1 | 2 | 3 |
| **6.** Becoming easily annoyed or irritable | 0 | 1 | 2 | 3 |
| **7.** Feeling afraid as if something awful might happen | 0 | 1 | 2 | 3 |

***(For office coding: Total Score T____ = ____ + ____ + ____ )***

Scheim and Bauer, 2019
https://doi.org/10.1016/j.socscimed.2018.12.016

## Intersectional Major Discrimination Index (InDI-M)

As a reminder, we are interested in experiences related to **who you are**. This includes both how you describe yourself and how others might describe you. For example, your skin color, ancestry, nationality, religion, gender, sexuality, age, weight, disability or mental health issue, and income.

1. Because of who you are, has a health care provider ever refused you care?

   ☐ Never → Skip to 2
   ☐ Once
   ☐ More than once

   1a. Has this happened to you in the past 12 months?
   ☐ Yes
   ☐ No

2. Because of who you are, have you ever been fired or dismissed from a job, or been turned down for a job that you interviewed for?

   ☐ Never → Skip to 3
   ☐ Once
   ☐ More than once

   2a. Has this happened to you in the past 12 months?
   ☐ Yes
   ☐ No

3. Because of who you are, have you ever been evicted or denied housing?

   ☐ Never → Skip to 4
   ☐ Once
   ☐ More than once

   3a. Has this happened to you in the past 12 months?
   ☐ Yes
   ☐ No

4. Because of who you are, have you ever been unreasonably stopped and questioned, searched, or arrested by police or security?

   ☐ Never → Skip to 5
   ☐ Once
   ☐ More than once

   4a. Has this happened to you in the past 12 months?
   ☐ Yes
   ☐ No

5. Because of who you are, have you ever been unreasonably expelled or suspended from school?

   ☐ Never → Skip to 6
   ☐ Once
   ☐ More than once

   5a. Has this happened to you in the past 12 months?
   ☐ Yes
   ☐ No

6. Because of who you are, have you ever been unable to open a bank account, cash a cheque, or get a loan?

   ☐ Never → Skip to 7
   ☐ Once
   ☐ More than once

   6a. Has this happened to you in the past 12 months?
   ☐ Yes
   ☐ No

7. Because of who you are, have you ever had to move to another neighborhood, town, city, state, province, or country?

   ☐ Never → Skip to 8
   ☐ Once
   ☐ More than once

   7a. Has this happened to you in the past 12 months?
   ☐ Yes
   ☐ No

8. Because of who you are, have you ever lost a close relationship (e.g., with a family member, friend, or partner)?

   ☐ Never → Skip to 9
   ☐ Once
   ☐ More than once

   8a. Has this happened to you in the past 12 months?
   ☐ Yes
   ☐ No

9. Because of who you are, have you ever been repeatedly harassed at work or school, where you live, or when accessing services?

   ☐ No → Skip to 10
   ☐ Yes—in one place
   ☐ Yes—in more than one place

   9a. Has this happened to you in the past 12 months?
   ☐ Yes
   ☐ No

10. Because of who you are, have you ever been threatened with a physical or sexual attack?

   ☐ Never → Skip to 11
   ☐ Once
   ☐ More than once

   10a. Has this happened to you in the past 12 months?
   ☐ Yes
   ☐ No

11. Because of who you are, have you ever been physically attacked (e.g., spit on, had objects thrown at you, hit, punched, pushed or grabbed, beaten)?

   ☐ Never → Skip to 12
   ☐ Once

☐ More than once

11a. Has this happened to you in the past 12 months?
☐ Yes
☐ No

12. Because of who you are, have you ever been made to engage in sexual activity, or been touched in a sexual way, that you didn't want?

☐ Never → Skip to 13
☐ Once
☐ More than once

12a. Has this happened to you in the past 12 months?
☐ Yes
☐ No

13. Because of who you are, have you ever had someone take, damage, or vandalize your property?

☐ Never → Skip to next section
☐ Once
☐ More than once

13a. Has this happened to you in the past 12 months?
☐ Yes
☐ No

## SCORING

**Note:** Please see Scheim & Bauer 2019 for rationale. These are novel measures that continue to be evaluated in ongoing studies. You are free to modify response options and scoring procedures. We welcome feedback or observations on your experience using these measures.

**InDI-M.**
Lifetime major discrimination: for each item (1-13), code as 0 (never), 1 (once), or 2 (more than once; or in more than one place for Item 9). Sum to generate frequency scores ranging from 0-26.

Past year major discrimination: for each item (1a-13a), code as 1 (yes) or 0 (no). Sum to generate frequency scores ranging from 0-13.

**Cognitive emotion regulation questionnaire (CERQ)**

Everyone experiences negative or unpleasant events at some point in their lives, and each person reacts to them in their own way. The following questions ask about what you generally think when you experience negative or unpleasant events.

**"When I experience negative or unpleasant events..."**

| | Almost never, | Sometimes | Regularly | Often | Almost always |
|---|---|---|---|---|---|
| I feel that I am the one who is responsible for what has happened | | | | | |
| I think that basically the cause must lie within myself | | | | | |
| I think that I have to accept the situation | | | | | |
| I think that I cannot change anything about it | | | | | |
| I am preoccupied with what I think and feel about what I have experienced | | | | | |
| I dwell upon the feelings the situation has evoked in me | | | | | |
| I think of pleasant things that have nothing to do with it | | | | | |
| I think of something nice instead of what has happened | | | | | |
| I think about how I can best cope with the situation | | | | | |
| I think about a plan of what I can do best | | | | | |
| I think that I can become a stronger person as a result of what has happened | | | | | |
| I look for the positive sides to the matter | | | | | |
| I think that other people go through much worse experiences | | | | | |
| I tell myself that there are worse things in life | | | | | |
| I often think that what I have experienced is much worse than what others have experienced | | | | | |
| I often think that what I have experienced is the worst that can happen to a person | | | | | |
| I feel that others are to blame for it | | | | | |
| I think about the mistakes others have made in this matter | | | | | |

# Emotion Regulation Questionnaire

**Name:** ________________________________________ **Date:** _____________

**Instructions:** Please check the corresponding number that indicates how much you agree with each item by using the following scale:

**1 =** Strongly Disagree

**2 =** Disagree

**3 =** Slightly Disagree

**4 =** Neutral

**5 =** Slightly Agree

**6 =** Agree

**7 =** Strongly Agree

| Statement | 1 | 2 | 3 | 4 | 5 | 6 | 7 |
|---|---|---|---|---|---|---|---|
| 1. When I want to feel more positive emotions (such as joy or amusement), I change my thoughts. | ☐ | ☐ | ☐ | ☐ | ☐ | ☐ | ☐ |
| 2. I keep my emotions to myself. | ☐ | ☐ | ☐ | ☐ | ☐ | ☐ | ☐ |
| 3. When I'm faced with a stressful situation, I think about it in a way that helps me stay calm. | ☐ | ☐ | ☐ | ☐ | ☐ | ☐ | ☐ |
| 4. I control my emotions by not expressing them. | ☐ | ☐ | ☐ | ☐ | ☐ | ☐ | ☐ |
| 5. When I want to feel less negative emotion (such as sadness or anger), I change my thoughts. | ☐ | ☐ | ☐ | ☐ | ☐ | ☐ | ☐ |
| 6. I control my emotions by changing the way I think about the situation I'm in. | ☐ | ☐ | ☐ | ☐ | ☐ | ☐ | ☐ |

| | | | | | | | |
|---|---|---|---|---|---|---|---|
| 7. When feeling positive emotions, I am careful not to express them. | ☐ | ☐ | ☐ | ☐ | ☐ | ☐ | ☐ |
| 8. When I'm faced with a situation that could upset me, I think about it in a way that helps me stay calm. | ☐ | ☐ | ☐ | ☐ | ☐ | ☐ | ☐ |
| 9. I control my emotions by changing what I think about. | ☐ | ☐ | ☐ | ☐ | ☐ | ☐ | ☐ |
| 10. When I feel negative emotions, I make sure not to express them. | ☐ | ☐ | ☐ | ☐ | ☐ | ☐ | ☐ |
| **Score:** | | | | | | | |

**Score interpretation:**

Items 1, 3, 5, 6, 8, and 9 assess cognitive reappraisal. Items 2, 4, 7, and 10 assess expressive suppression.

For cognitive reappraisal and expressive suppression, scores can range from 6 to 42 and 4 to 28, respectively. A higher score indicates greater use of that emotion regulation strategy.

1. **Cognitive Reappraisal:** This form of cognitive-emotional regulation involves changing the way one thinks about potentially emotion-eliciting events to alter their emotional impact. Essentially, it is a way of reinterpreting a situation to decrease its emotional impact. For example, if someone makes a rude comment to you, rather than letting it upset you, you might tell yourself that the person is just having a bad day and is taking it out on others, which has nothing to do with you.

2. **Expressive Suppression:** This is another emotion regulation strategy that involves inhibiting or reducing the external signs of your inner emotional state. It's concealing your emotions, like keeping a poker face. For instance, if you're feeling upset during a meeting at work, you might try to suppress any signs of this emotion by maintaining a neutral facial expression.

Remember, these are not necessarily 'good' or 'bad' strategies. The effectiveness of either strategy can depend on a range of factors, including the specific situation, the intensity of the emotion, the individual's overall emotional profile, and cultural norms. Also, flexibly employing a range of strategies as needed is often more beneficial than relying on any one strategy.

NovoPsych

# Brief - Coping Orientation to Problems Experienced Inventory (Brief-COPE)

## Instructions:

The following questions ask how you have sought to cope with a hardship in your life. Read the statements and indicate how much you have been using each coping style.

| | | I haven't been doing this at all | A little bit | A medium amount | I’ve been doing this a lot |
|---|---|---|---|---|---|
| 1 | I've been turning to work or other activities to take my mind off things. | **1** | **2** | **3** | **4** |
| 2 | I've been concentrating my efforts on doing something about the situation I'm in. | **1** | **2** | **3** | **4** |
| 3 | I've been saying to myself "this isn't real". | **1** | **2** | **3** | **4** |
| 4 | I've been using alcohol or other drugs to make myself feel better | **1** | **2** | **3** | **4** |
| 5 | I've been getting emotional support from others. | **1** | **2** | **3** | **4** |
| 6 | I've been giving up trying to deal with it. | **1** | **2** | **3** | **4** |
| 7 | I've been taking action to try to make the situation better. | **1** | **2** | **3** | **4** |
| 8 | I've been refusing to believe that it has happened. | **1** | **2** | **3** | **4** |
| 9 | I've been saying things to let my unpleasant feelings escape. | **1** | **2** | **3** | **4** |
| 10 | I’ve been getting help and advice from other people. | **1** | **2** | **3** | **4** |
| 11 | I've been using alcohol or other drugs to help me get through it. | **1** | **2** | **3** | **4** |
| 12 | I've been trying to see it in a different light, to make it seem more positive. | **1** | **2** | **3** | **4** |
| 13 | I’ve been criticizing myself. | **1** | **2** | **3** | **4** |
| 14 | I've been trying to come up with a strategy about what to do. | **1** | **2** | **3** | **4** |
| 15 | I've been getting comfort and understanding from someone. | **1** | **2** | **3** | **4** |
| 16 | I've been giving up the attempt to cope. | **1** | **2** | **3** | **4** |

NovoPsych

| | | I haven't been doing this at all | A little bit | A medium amount | I've been doing this a lot |
|---|---|---|---|---|---|
| 17 | I've been looking for something good in what is happening. | 1 | 2 | 3 | 4 |
| 18 | I've been making jokes about it. | 1 | 2 | 3 | 4 |
| 19 | I've been doing something to think about it less, such as going to movies, watching TV, reading, daydreaming, sleeping, or shopping. | 1 | 2 | 3 | 4 |
| 20 | I've been accepting the reality of the fact that it has happened. | 1 | 2 | 3 | 4 |
| 21 | I've been expressing my negative feelings. | 1 | 2 | 3 | 4 |
| 22 | I've been trying to find comfort in my religion or spiritual beliefs. | 1 | 2 | 3 | 4 |
| 23 | I've been trying to get advice or help from other people about what to do. | 1 | 2 | 3 | 4 |
| 24 | I've been learning to live with it. | 1 | 2 | 3 | 4 |
| 25 | I've been thinking hard about what steps to take. | 1 | 2 | 3 | 4 |
| 26 | I've been blaming myself for things that happened | 1 | 2 | 3 | 4 |
| 27 | I've been praying or meditating | 1 | 2 | 3 | 4 |
| 28 | I've been making fun of the situation. | 1 | 2 | 3 | 4 |

## Developer Reference:

Carver, C. S. (1997). You want to measure coping but your protocol’too long: Consider the brief cope. International journal of behavioral medicine, 4(1), 92-100.

Administer Now

Multidimensional Scale of Perceived Social Support

Instructions: We are interested in how you feel about the following statements. Read each statement carefully. Indicate how you feel about each statement.

Circle the "1" if you **Very Strongly Disagree**
Circle the "2" if you **Strongly Disagree**
Circle the "3" if you **Mildly Disagree**
Circle the "4" if you are **Neutral**
Circle the "5" if you **Mildly Agree**
Circle the "6" if you **Strongly Agree**
Circle the "7" if you **Very Strongly Agree**

| | Very Strongly Disagree | Strongly Disagree | Mildly Disagree | Neutral | Mildly Agree | Strongly Agree | Very Strongly Agree |
|---|---|---|---|---|---|---|---|
| 1. There is a special person who is around when I am in need. | 1 | 2 | 3 | 4 | 5 | 6 | 7 |
| 2. There is a special person with whom I can share joys and sorrows. | 1 | 2 | 3 | 4 | 5 | 6 | 7 |
| 3. My family really tries to help me. | 1 | 2 | 3 | 4 | 5 | 6 | 7 |
| 4. I get the emotional help & support I need from my family. | 1 | 2 | 3 | 4 | 5 | 6 | 7 |
| 5. I have a special person who is a real source of comfort to me. | 1 | 2 | 3 | 4 | 5 | 6 | 7 |
| 6. My friends really try to help me. | 1 | 2 | 3 | 4 | 5 | 6 | 7 |
| 7. I can count on my friends when things go wrong. | 1 | 2 | 3 | 4 | 5 | 6 | 7 |
| 8. I can talk about my problems with my family. | 1 | 2 | 3 | 4 | 5 | 6 | 7 |
| 9. I have friends with whom I can share my joys and sorrows. | 1 | 2 | 3 | 4 | 5 | 6 | 7 |
| 10. There is a special person in my life who cares about my feelings. | 1 | 2 | 3 | 4 | 5 | 6 | 7 |
| 11. My family is willing to help me make decisions. | 1 | 2 | 3 | 4 | 5 | 6 | 7 |
| 12. I can talk about my problems with my friends. | 1 | 2 | 3 | 4 | 5 | 6 | 7 |

Scale Reference:

Zimet GD, Dahlem NW, Zimet SG, Farley GK. The Multidimensional Scale of Perceived Social Support. *Journal of Personality Assessment* 1988;52:30-41.

Scoring Information:

To calculate mean scores:

Significant Other Subscale: Sum across items 1, 2, 5, & 10, then divide by 4.

Family Subscale: Sum across items 3, 4, 8, & 11, then divide by 4.

Friends Subscale: Sum across items 6, 7, 9, & 12, then divide by 4.

Total Scale: Sum across all 12 items, then divide by 12.

More information at:

http://gzimet.wix.com/mspss

Other MSPSS Scoring Options:

There are no established population norms on the MSPSS. Also, norms would likely vary on the basis of culture and nationality, as well as age and gender. I have typically looked at how social support differs between groups (e.g., married compared to unmarried individuals) or is associated with other measures (e.g., depression or anxiety). With these approaches you can use the mean scale scores.

If you want to divide your respondents into groups on the basis of MSPSS scores there are at least two ways you can approach this process:

1. You can divide your respondents into 3 equal groups on the basis of their scores (trichotomize) and designate the lowest group as low perceived support, the middle group as medium support, and the high group as high support. This approach ensures that you have about the same number of respondents in each group. But, if the distribution of scores is skewed, your low support group, for example, may include respondents who report moderate or even relatively high levels of support.

2. Alternatively, you can use the scale response descriptors as a guide. In this approach any mean scale score ranging from 1 to 2.9 could be considered low support; a score of 3 to 5 could be considered moderate support; a score from 5.1 to 7 could be considered high support. This approach would seem to have more validity, but if you have very few respondents in any of the groups, it could be problematic.
